# Clipping of Bilateral Ophthalmic Artery Aneurysms Through a Single Craniotomy

**DOI:** 10.7759/cureus.47291

**Published:** 2023-10-18

**Authors:** Jhon E Bocanegra-Becerra, Stefan W Koester, Sávio Batista, Caio M Perret, Raphael Bertani

**Affiliations:** 1 Faculty of Medicine, Universidad Peruana Cayetano Heredia, Lima, PER; 2 Medical School, Vanderbilt University School of Medicine, Nashville, USA; 3 Faculty of Medicine, Federal University of Rio de Janeiro, Rio de Janeiro, BRA; 4 Neurological Surgery, Hospital Municipal Miguel Couto, Rio de Janeiro, BRA; 5 Neuroscience, Federal University of Rio de Janeiro, Rio de Janeiro, BRA; 6 Neurosurgery, University of São Paulo, São Paulo, BRA

**Keywords:** operative video, bilateral ophthalmic aneurysms, microsurgery, paraclinoid aneurysm, ophthalmic aneurysm

## Abstract

Bilateral ophthalmic aneurysms are rare and involve two aneurysms in the ophthalmic arteries, one on each, leading to potential symptoms such as vision loss and headaches. The treatment options for aneurysms, ranging from surgery and endovascular embolization to observation, depend on various factors, including aneurysm size and the patient's health. Microsurgery, while presenting complexities due to the intricate anatomy of the anterior clinoid region, offers potential advantages such as enhanced decompression rates and reduced aneurysm recurrence. The presented surgical video illustrates the treatment of bilateral ophthalmic artery aneurysms via a single craniotomy. This method reduces surgical duration and trauma, facilitating quicker patient recovery. However, this method bears potential risks, especially to both optic nerves. As underscored in the video, the utmost anatomical understanding in the anterior clinoid area is pivotal for successful outcomes and reduced complications.

## Introduction

Bilateral ophthalmic aneurysms are rare conditions in which two aneurysms are located in the ophthalmic artery, a branch of the internal carotid artery that supplies blood to the eye and surrounding structures. According to Hong and Wang, patients presenting with intracranial aneurysms frequently exhibit multiple aneurysms, ranging from 7% to 34% of cases [1]. Additionally, bilateral aneurysms are observed in a substantial subset of these patients, specifically between 20% and 40%. These aneurysms can cause a variety of symptoms, including vision loss, headaches, and eye pain, and they can be treated with surgery, endovascular embolization, or observation. The risk factors, symptoms, and treatment options for bilateral ophthalmic aneurysms will depend on the size and location of the aneurysms and the overall health of the patient [2].

Microsurgery for ophthalmic aneurysms is often challenging because of the complex anatomy surrounding the affected vessel in the anterior clinoid region [3-5]. Moreover, the microsurgical treatment of these lesions has been proven to allow for greater decompression rates by the mass effect and less risk of aneurysm recurrence [3,6,7]. As a result, careful planning and vast experience from the neurosurgeon are needed to optimize patient outcomes in challenging scenarios such as in the case of bilateral aneurysm presentations.

Here, we present a surgical video where was performed a single craniotomy for bilateral clipping of ophthalmic artery aneurysms, detailing operative technique and nuances.

## Technical report

A 54-year-old female presented to the emergency department complaining of an acute, severe headache and right-sided visual loss. She was alert and oriented, without any motor deficits, but could only perceive shadows with her right eye. CT angiography showed bilateral ophthalmic artery aneurysms. The patient underwent microsurgical treatment for both aneurysms through a single pterional craniotomy, and complete occlusion of the aneurysms was proved by indocyanine green imaging.

Postoperatively, the previous right-side vision loss persisted without the addition of neurological deficits. Video 1 provides a detailed view of the surgery.

**Video 1:** Clipping of bilateral ophthalmic artery aneurysms.

## Discussion

A single craniotomy to treat bilateral aneurysms is a surgical procedure in which a single incision is made on the skull to access and treat two aneurysms located on opposite sides of the brain. This approach can be used if the aneurysms are located in close proximity to each other and can be accessed through the same incision. The advantages of using a single craniotomy to treat bilateral aneurysms include reduced surgical time, less trauma to the brain, and a shorter recovery period [8]. 

By performing a single craniotomy on both sides of the brain, the surgeon can address the aneurysms on both sides in one sitting, reducing the overall surgical time and minimizing the need for multiple surgeries. Furthermore, with a single craniotomy, patients typically have a faster recovery time and can return to normal activities sooner than with multiple surgeries, improving the chances of a long-term positive outcome [1]. These advantages make single craniotomy a feasible option to treat bilateral aneurysms.

However, this approach for bilateral ophthalmic artery treatment may not be suitable for all patients and the decision to use this approach will depend on the location and size of the aneurysms, as well as the patient's overall health. The major risk of a single operation for bilateral ophthalmic segment aneurysms is the risk to both optic nerves. Visual loss affecting both nerves is potentially devastating. In this experience, only one patient had a vision change related to surgery, and this occurred in the eye ipsilateral to the craniotomy. Anterior clinoidectomy appears to be the greatest risk to vision, due to drilling the clinoid process, injury to small perforators on the optic nerve, direct manipulation during the dissection of the distal dural rings, or a combination of these factors [9].

## Conclusions

Therefore, a planned surgery and knowledge of anatomy are necessary to perform a safe procedure with a reduced risk of complications. Surgical videos detailing the procedure can support this planning and, consequently, improve patient clinical outcomes. Overall, this operative video demonstrates a step-wise approach to clipping bilateral ophthalmic aneurysms via a single craniotomy and highlights the importance of anatomical knowledge in the anterior clinoid region.
